# Supplementary material for: Lysosomal integral membrane protein-2 (LIMP-2/SCARB2) is involved in lysosomal cholesterol export
Source: Nat Commun. 2019 Aug 6;10:3521. doi: 10.1038/s41467-019-11425-0 (PMC6684646; doi:10.1038/s41467-019-11425-0)
Supplement: Supplementary file 1 — Supplementary Information [file 41467_2019_11425_MOESM1_ESM.pdf]

Supplementary information

Lysosomal Integral Membrane Protein-2 (LIMP-2/SCARB2) is involved in lysosomal cholesterol export

Heybrock, Kanerva, Meng et al

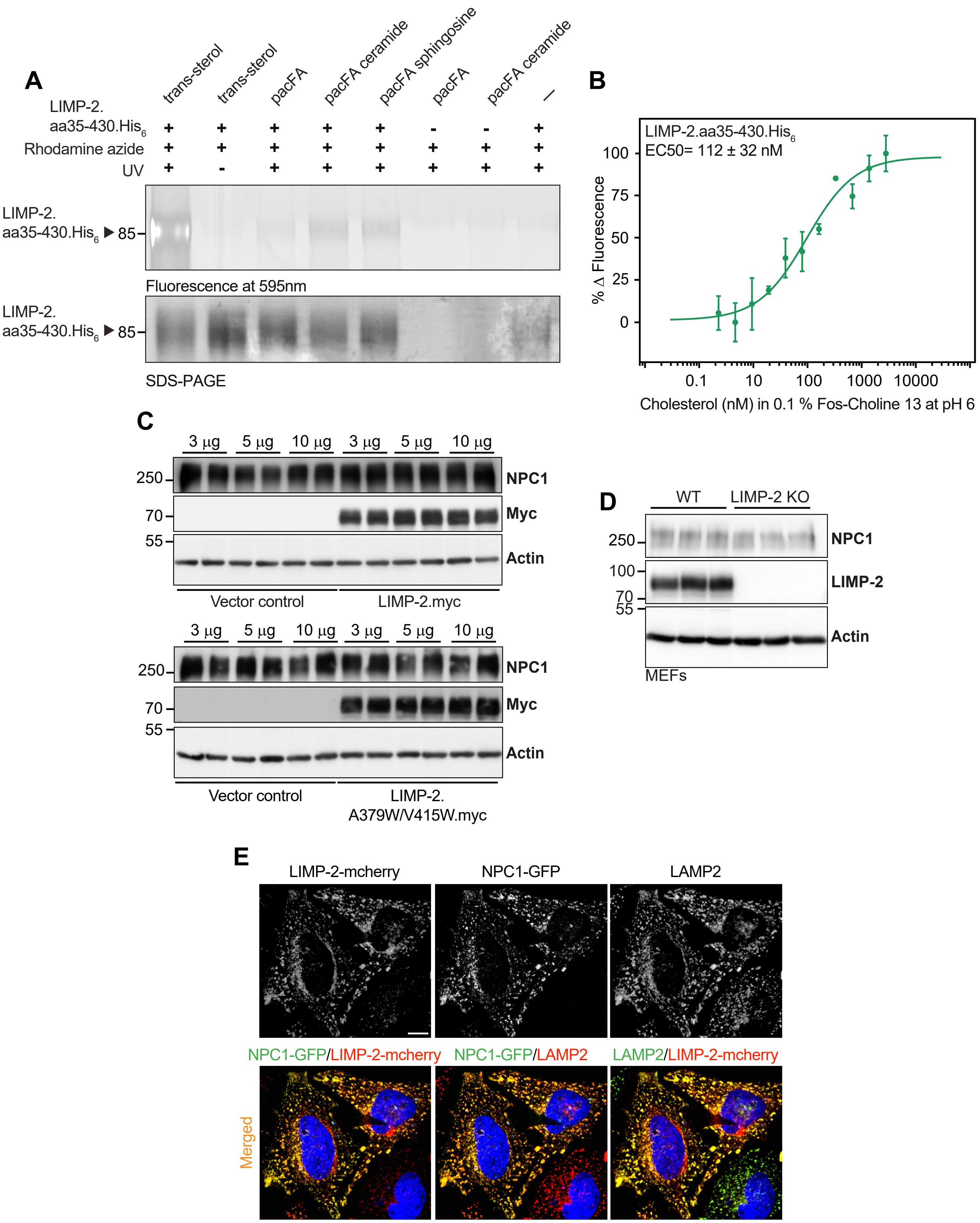

A
trans-sterol
trans-sterol
pacFA
pacFA ceramide
pacFA sphingosine
pacFA
pacFA ceramide
LIMP-2.
aa35-430.His6
Rhodamine azide
UV
85
Fluorescence at 595nm
SDS-PAGE
B
LIMP-2.aa35-430.His6
EC50= 112 ± 32 nM
% Δ Fluorescence
Cholesterol (nM) in 0.1 % Fos-Choline 13 at pH 6
C
3 µg
5 µg
10 µg
NPC1
Myc
Actin
Vector control
LIMP-2.myc
LIMP-2.
A379W/V415W.myc
D
WT
LIMP-2 KO
LIMP-2
MEFs
E
LIMP-2-mcherry
NPC1-GFP
LAMP2
NPC1-GFP/LIMP-2-mcherry
NPC1-GFP/LAMP2
LAMP2/LIMP-2-mcherry
Merged

Supplementary Figure 1: Gel-based profiling of photoclickable lipid probes and microscale thermophoresis reveal direct binding of cholesterol to the ectodomain of LIMP-2

(**A**) Lower panel: SDS-PAGE analysis of purified LIMP-2 ectodomain (LIMP-2.35-430.His$_6$) treated with 50 µM of photoclickable lipid probes with or without 365-nm UV radiation before click chemistry. Upper panel:in-gel fluorescence scanning. Trans-sterol, Hex-5'-ynyl 3ß-hydroxy-6-diazirinyl-5α-cholan-24-oate; pacFA, 9-(3-pent-4-ynyl-3-H-diazirin-3-yl)-nonanoic acid; pacFA sphingosine, (2S,3R,E)-2-amino-13-(3-(pent-4-yn-1-yl)-3H-diazirin-3-yl)tridec-4-ene-1,3-diol; pacFA ceramide, N-(9-(3-pent-4-ynyl-3-H-diazirin-3-yl)-nonanoyl)-D-erythro-sphingosine. (**B**) Effect of cholesterol on fluorescence decay of Red-tris-NTA labeled LIMP-2 protein using microscale thermophoresis (n = 3). (**C**) Effect of myc-tagged LIMP-2-WT overexpression on NPC1 stability using different amounts of plasmids. The stability of the endogenous NPC1 and the expression of the myc-tagged LIMP-2 were followed by immunoblot. (**D**) Immunoblot analysis of NPC1, LIMP-2 and actin expression in LIMP-2 knockout MEFs compared to wildtype (WT) MEFs. (**E**) Confocal microscopy of HeLa cells transiently co-expressing C-terminally GFP-tagged wild-type NPC1 (NPC1.GFP) and C-terminally mCherry-tagged wild-type LIMP-2 (LIMP-2.mCherry). Lysosomes were stained with LAMP-2. Scale bar: 10 µm. Source data are provided as a Source Data file.

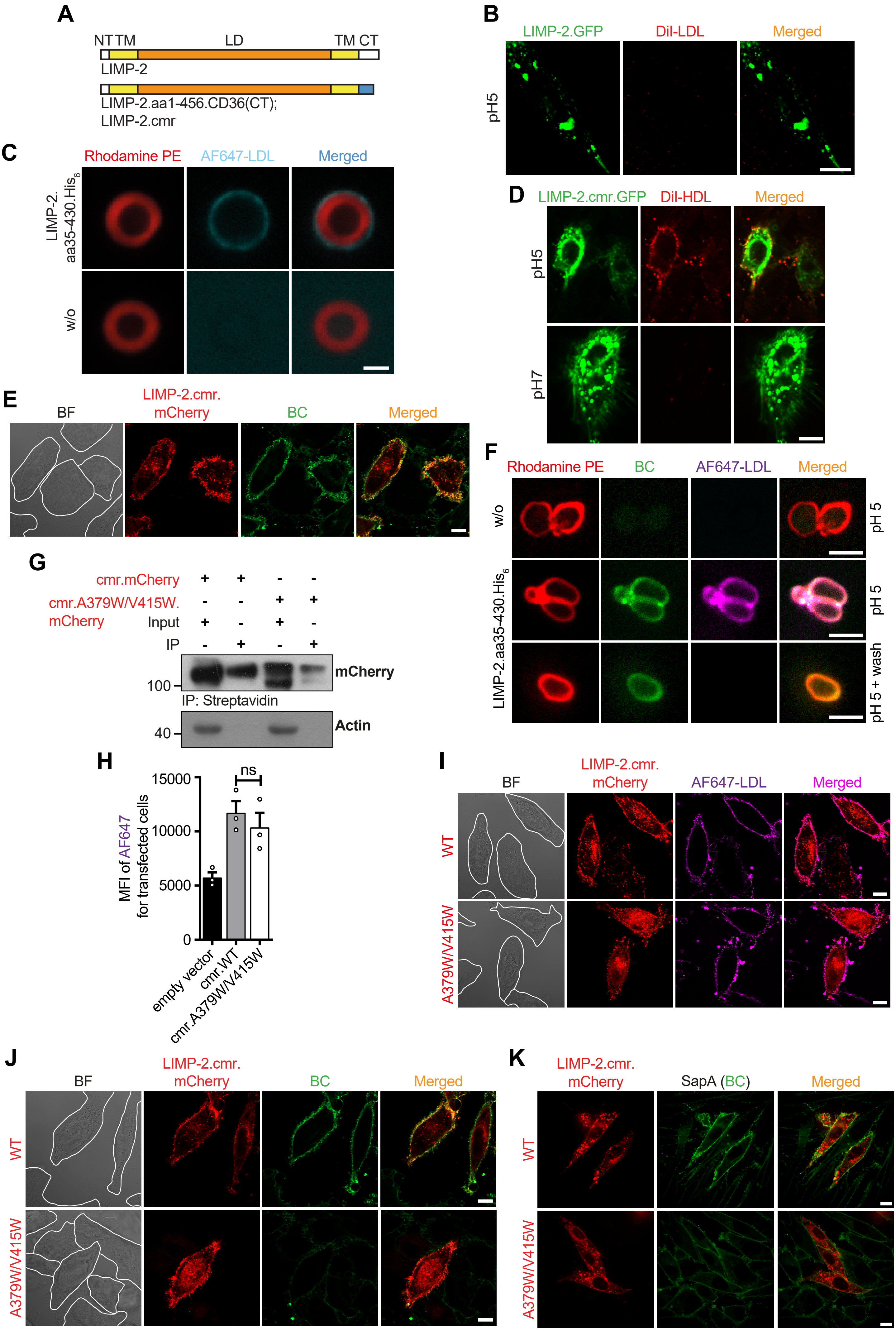

A
NT TM
LD
TM CT
LIMP-2
LIMP-2.aa1-456.CD36(CT);
LIMP-2.cmr
B
LIMP-2.GFP
DiI-LDL
Merged
pH5
C
Rhodamine PE
AF647-LDL
Merged
LIMP-2. aa35-430.His6
w/o
D
LIMP-2.cmr.GFP
DiI-HDL
Merged
pH5
pH7
E
BF
LIMP-2.cmr. mCherry
BC
Merged
F
Rhodamine PE
BC
AF647-LDL
Merged
w/o
pH 5
LIMP-2.aa35-430.His6
pH 5
pH 5 + wash
G
cmr.mCherry
cmr.A379W/V415W. mCherry
Input
IP
mCherry
100
IP: Streptavidin
Actin
40
H
MFI of AF647 for transfected cells
15000
10000
5000
0
ns
empty vector
cmr.WT
cmr.A379W/V415W
I
BF
LIMP-2.cmr. mCherry
AF647-LDL
Merged
WT
A379W/V415W
J
BF
LIMP-2.cmr. mCherry
BC
Merged
WT
A379W/V415W
K
LIMP-2.cmr. mCherry
SapA (BC)
Merged
WT
A379W/V415W

Supplementary Figure 2: Lipoprotein binding to LIMP-2 ectodomain

(A) Characterization of the plasma membrane-expressing LIMP-2 chimeric construct, LIMP-2-cmr. Schematic representation of the LIMP-2 chimeric construct in which the cytoplasmic C-terminus of LIMP-2 (aa 457-478) was substituted with the cytoplasmic C-terminal fragment of CD36 (aa 463-472; shown in blue). (**B**) Confocal images of CHO cells transiently expressing LIMP-2.GFP showing no binding of DiI-LDL at pH 5. Scale bars, 10 µm. (**C**) Confocal images of succinimidyl ester-labeled LDL binding to recombinant LIMP-2 ectodomain (LIMP-2.aa35-430.His_6) tethered on fluorescently-labeled liposomes. Lower panel represents the negative control in which LIMP-2.aa35-430.His_6 protein is not present. Scale bar, 10 µm. (**D**) Confocal microscopy of DiI-HDL binding to CHO cells expressing GFP-tagged chimeric LIMP-2 (LIMP-2.cmr.GFP) at acidic and neutral pH. Scale bar, 10 µm. (**E**) Confocal images of BC labeling derived from AF647-LDL(BC) in HeLa cells transiently expressing C-terminally mCherry-tagged chimeric LIMP-2 (LIMP-2.cmr.mCherry). Cells treated as in Fig. 2E (upper panel) were further washed with HBSS. (**F**) Confocal images of BC labeling derived from (AF647-LDL(BC) using recombinant LIMP-2 ectodomain (LIMP-2.aa35-430.His_6) tethered on fluorescently-labeled liposomes. The upper panel represents the negative control in which LIMP-2.aa35-430.His_6 protein is not present. The lower panel represents the BC uptake mediated by LIMP-2 after LDL washing with PBS. Scale bars, 10 µm. (**G**) Plasmalemmal expression of chimeric C-terminally mCherry-tagged LIMP-2.cmr and LIMP-2.cmr.A379W/V415W transiently transfected in HeLa cells was detected using Western blotting of biotinylated cell surface proteins after immunoprecipitation with streptavidin-beads. (**H**, **I**) Flow cytometry analysis (1000 transfected cells/group; n= 3 experiments) (**H**) and confocal images (**I**) of Alexa Fluor 647 succinimidyl ester-labeled LDL binding to CHO (**H**) or HeLa cells (**I**), respectively, transiently expressing mCherry-tagged-wild type (LIMP-2.cmr.mCherry) or tunnel-blocking mutant form of LIMP-2 chimera (LIMP-2.cmr.A379W/V415W.mCherrry). (**J**) Confocal microscopy of BC labeling derived from AF647-LDL(BC)-derived in HeLa cells expressing LIMP-2.cmr.mCherry or LIMP-2.cmr.A379W/V415W.mCherry proteins. Cells were pre-incubated with doubly labelled LDL (AF647-LDL(BC)) and washed with HBSS. (**K**) Confocal microscopy of BC labeling derived from SapA(BC) picodisks in CHO cells transiently expressing C-terminally mCherry-tagged-wild type (LIMP-2.cmr.mCherry) or tunnel-blocking mutant LIMP-2 chimera (LIMP-2.cmr.A379W/V415W.mCherrry). Cells transiently expressing LIMP-2.cmr.mCherry were incubated with SapA(BC) picodiscs at acidic pH followed by washing with PBS. Scale bars, 10 µm. Data are presented as mean ± SEM. Statistical analyses were performed using an unpaired two-tailed Student's t-test. Source data are provided as a Source Data file.

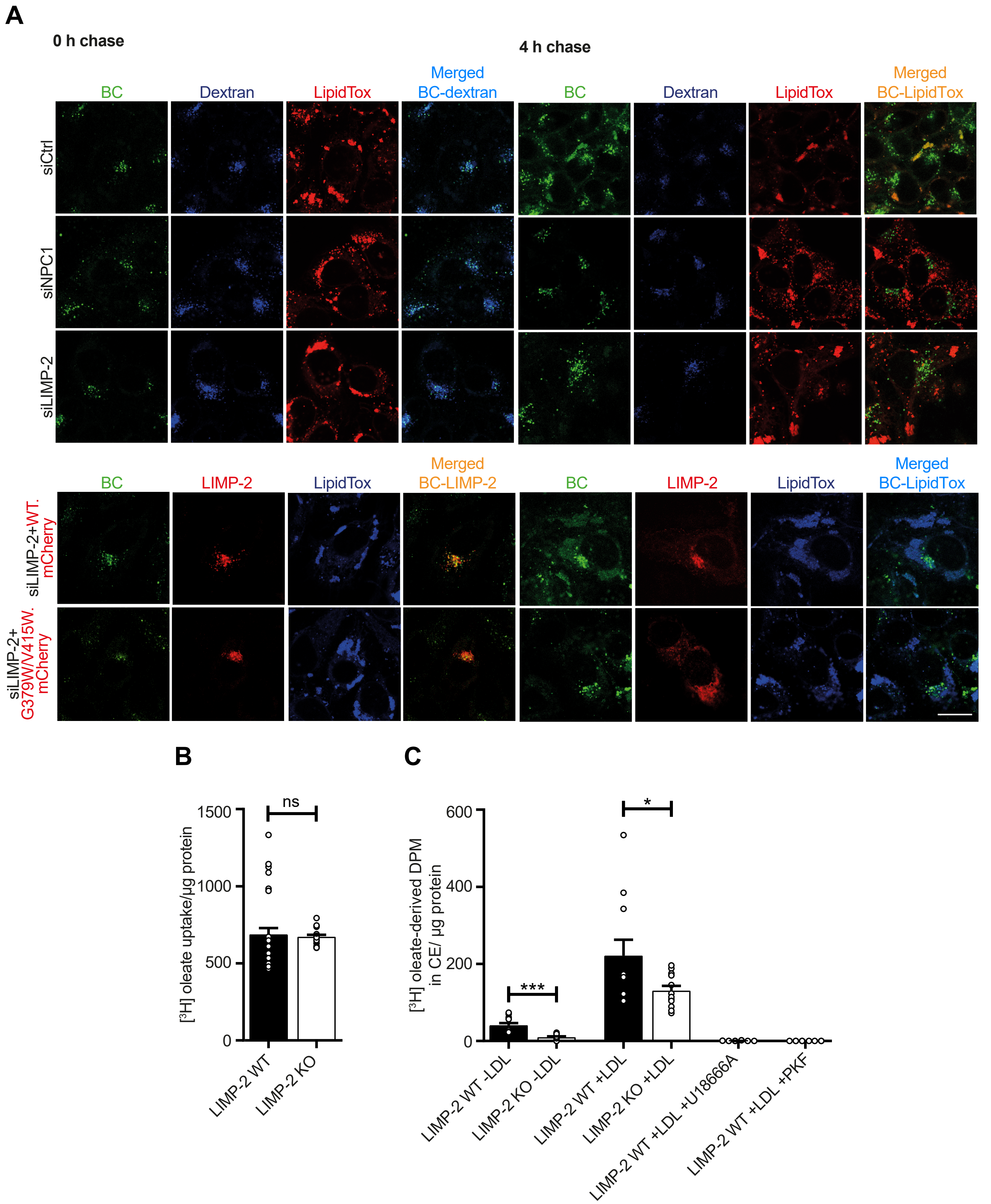

Supplementary Figure 3: Live cell tracking of labelled cholesterol to lipid droplets

(**A**) Single-channel confocal images of BC, dextran, LipidTox and LIMP-2-WT/-TM (tunnel mutant) in the A431 cells shown in Fig. 3B. Scale bar, 10 µm. (**B**) Uptake of [^{3}H]oleate into LIMP-2-WT and -KO MEFs (n =18 [WT], n=30 [KO] independent samples from three experiments, mean ± SEM). (**C**) Analysis of [^{3}H]oleate incorporation into cholesteryl esters in LIMP-2-WT and -KO MEFs in the presence and absence of LDL and U18666A or PKF. n=11 (-LDL), n=12 (+LDL), n=6 (PKF/U18666A) independent samples. Data (mean ± SEM) from two (for U18666A and PKF) to three (for LIMP-2 WT and KO +/- LDL) independent experiments. Statistical analyses were performed using an unpaired two-tailed Student's t-test (*P≤0.05, ***P≤0.001).

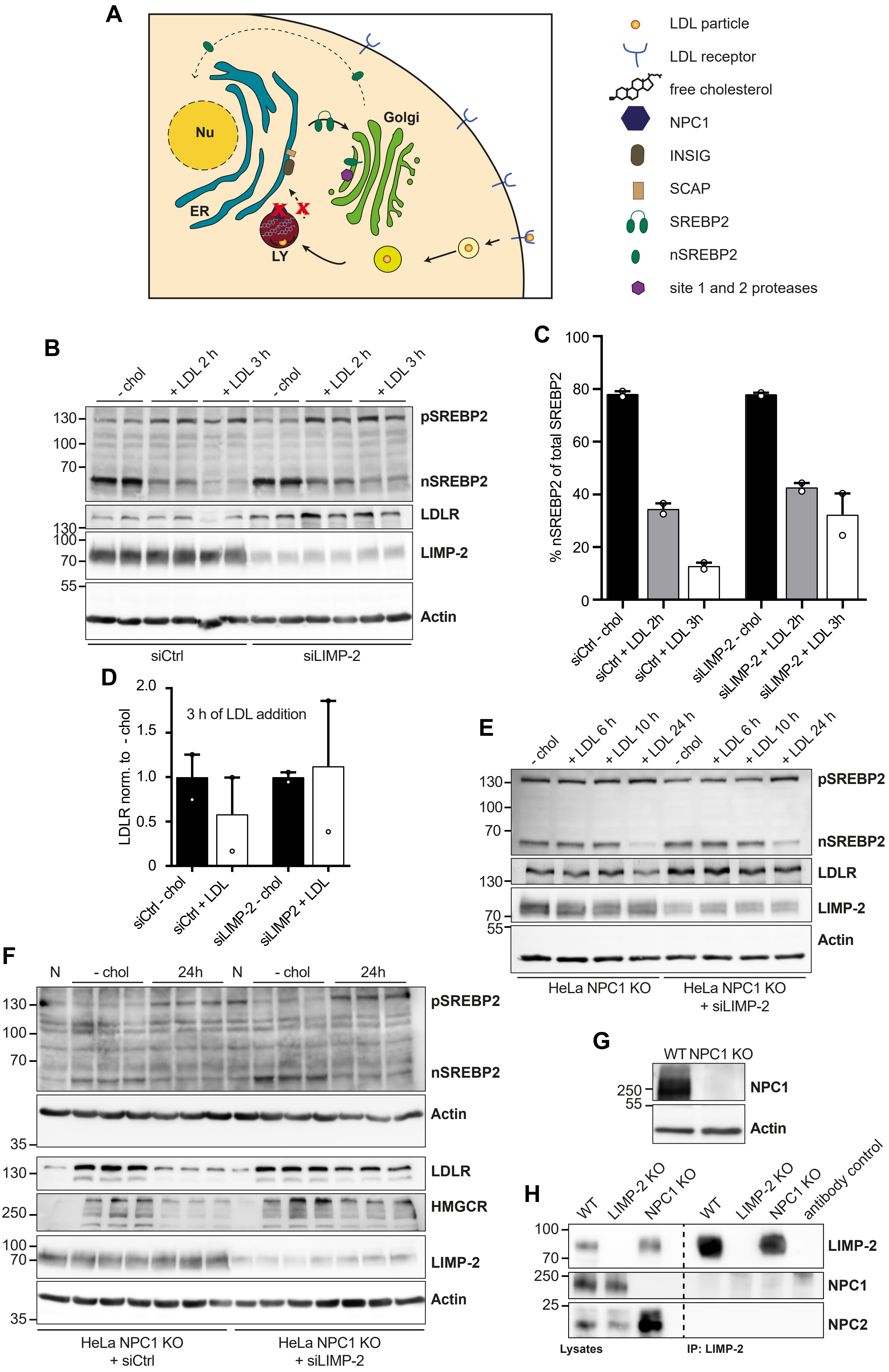
A
Nu
ER
Golgi
LY
LDL particle
LDL receptor
free cholesterol
NPC1
INSIG
SCAP
SREBP2
nSREBP2
site 1 and 2 proteases
B
- chol
+ LDL 2 h
+ LDL 3 h
- chol
+ LDL 2 h
+ LDL 3 h
pSREBP2
nSREBP2
LDLR
LIMP-2
Actin
130
100
70
130
100
70
55
siCtrl
siLIMP-2
C
% nSREBP2 of total SREBP2
0
20
40
60
80
100
siCtrl - chol
siCtrl + LDL 2h
siCtrl + LDL 3h
siLIMP-2 - chol
siLIMP-2 + LDL 2h
siLIMP-2 + LDL 3h
D
3 h of LDL addition
LDLR norm. to - chol
0
0.5
1.0
1.5
2.0
siCtrl - chol
siCtrl + LDL
siLIMP-2 - chol
siLIMP2 + LDL
E
- chol
+ LDL 6 h
+ LDL 10 h
+ LDL 24 h
- chol
+ LDL 6 h
+ LDL 10 h
+ LDL 24 h
130
100
70
130
70
55
pSREBP2
nSREBP2
LDLR
LIMP-2
Actin
HeLa NPC1 KO
HeLa NPC1 KO
+ siLIMP-2
F
N
- chol
24h
N
- chol
24h
130
100
70
35
130
250
100
70
35
pSREBP2
nSREBP2
Actin
LDLR
HMGCR
LIMP-2
Actin
HeLa NPC1 KO
+ siCtrl
HeLa NPC1 KO
+ siLIMP-2
G
WT
NPC1 KO
250
55
NPC1
Actin
H
WT
LIMP-2 KO
NPC1 KO
WT
LIMP-2 KO
NPC1 KO
antibody control
100
70
250
25
LIMP-2
NPC1
NPC2
Lysates
IP: LIMP-2

Supplementary Figure 4: LIMP-2-dependent changes in transcriptional response to cholesterol

(**A**) Schematic representation of sterol regulatory element binding protein 2 (SREBP2) cleavage in response to ER cholesterol levels. The transcription factor SREBP2 is usually bound in the ER by the proteins INSIG and SCAP. When cholesterol is depleted in the ER, SREBP2 is released and transported to the Golgi via COPII-coated vesicles, where it is processed and cleaved by two proteases (site 1 and site 2 proteases) to yield a nuclear fragment (nSREBP2). This fragment translocates to the nucleus where it activates the transcription of genes involved in uptake or endogenous synthesis of cholesterol, respectively (e.g. LDLR, HMG-COAR). (**B**) Delayed termination of SREBP2 processing in response to abundant cholesterol after 2 h and 3 h of re-addition of LDL cholesterol in LIMP-2-silenced HeLa cells but not in control siRNA-treated HeLa cells. The processing of SREBP2 and the expression of LDLR as a reaction to high or low cholesterol levels was analyzed by immunoblot. (**C**) Quantification of nSREBP2 levels (from B). Nuclear SREBP2 fragment as percentage of total SREBP2 is depicted. n=2 from one experiment. (**D**) Quantification of LDLR levels (from B) after cholesterol loading, normalized to the cholesterol-depleted samples. n=2 from one experiment. (**E**) Analysis of the effect of cholesterol depletion and re-addition of LDL cholesterol for 6 h, 10 h and 24 h in NPC1-deficient HeLa cells on nSREBP2 formation. Cells were either untreated or treated with LIMP-2-specific siRNA. The processing of SREBP2, as well as the expression of the LDL receptor (LDLR) and LIMP-2, were followed by immunoblot. (**F**) Independent repeat of experiment shown in Fig. 4C demonstrating the effect of LDL re-addition for 24 h after cholesterol depletion in NPC1- deficient and NPC1/LIMP-2 double-depleted HeLa cells. Additionally to nSREBP2 and LDLR levels, the protein level of HMGCR was monitored by immunoblot analysis. (**G**) Immunoblot of WT and NPC1-KO cell lines revealing the complete absence of NPC1 protein. These NPC1-deficient HeLa cells were used in the experiments shown in Figs. 4C, S4E,F. (**H**) Immunoprecipitation experiments in HeLa wildtype (WT), LIMP-2 Knockout (KO) and NPC1 Knockout (KO) cells using 2 mg of cell lysate for precipitation (IP) of LIMP-2. No co-precipitation of LIMP-2 with NPC1 or NPC2 could be detected. Data are presented as mean ± SEM. Source data are provided as a Source Data file.
