# Supplementary material for: Lysosomal integral membrane protein-2 (LIMP-2/SCARB2) is involved in lysosomal cholesterol export
Source: Nat Commun. 2019 Aug 6;10:3521. doi: 10.1038/s41467-019-11425-0 (PMC6684646; doi:10.1038/s41467-019-11425-0)
Supplement: Supplementary file 3 — Reporting Summary [file 41467_2019_11425_MOESM3_ESM.pdf]

nature research

Corresponding author(s): Saftig, Grinstein, Neculai, Ikonen

Last updated by author(s): 28/6/19

Reporting Summary

Nature Research wishes to improve the reproducibility of the work that we publish. This form provides structure for consistency and transparency in reporting. For further information on Nature Research policies, see Authors & Referees and the Editorial Policy Checklist.

Statistics

For all statistical analyses, confirm that the following items are present in the figure legend, table legend, main text, or Methods section.

n/a	Confirmed	
☐	☒	The exact sample size (*n*) for each experimental group/condition, given as a discrete number and unit of measurement
☐	☒	A statement on whether measurements were taken from distinct samples or whether the same sample was measured repeatedly
☐	☒	The statistical test(s) used AND whether they are one- or two-sided<br>*Only common tests should be described solely by name; describe more complex techniques in the Methods section.*
☒	☐	A description of all covariates tested
☒	☐	A description of any assumptions or corrections, such as tests of normality and adjustment for multiple comparisons
☐	☒	A full description of the statistical parameters including central tendency (e.g. means) or other basic estimates (e.g. regression coefficient) AND variation (e.g. standard deviation) or associated estimates of uncertainty (e.g. confidence intervals)
☐	☒	For null hypothesis testing, the test statistic (e.g. *F*, *t*, *r*) with confidence intervals, effect sizes, degrees of freedom and *P* value noted<br>*Give P values as exact values whenever suitable.*
☒	☐	For Bayesian analysis, information on the choice of priors and Markov chain Monte Carlo settings
☒	☐	For hierarchical and complex designs, identification of the appropriate level for tests and full reporting of outcomes
☒	☐	Estimates of effect sizes (e.g. Cohen's *d*, Pearson's *r*), indicating how they were calculated

Our web collection on statistics for biologists contains articles on many of the points above.

Software and code

Policy information about availability of computer code

Data collection	No software used
Data analysis	As given in the manuscript; open source plugins have been used

For manuscripts utilizing custom algorithms or software that are central to the research but not yet described in published literature, software must be made available to editors/reviewers. We strongly encourage code deposition in a community repository (e.g. GitHub). See the Nature Research guidelines for submitting code & software for further information.

Data

Policy information about availability of data

All manuscripts must include a data availability statement. This statement should provide the following information, where applicable:

- Accession codes, unique identifiers, or web links for publicly available datasets
- A list of figures that have associated raw data
- A description of any restrictions on data availability

As given in the manuscript: The data that support the findings of this study are available from the corresponding authors upon reasonable request. A reporting summary for this Article is available as a Supplementary Information file. The source data underlying Figs. and Supplementary Figs. are provided as a Source Data file as well uncropped and unprocessed scans of western blots.

Field-specific reporting

Please select the one below that is the best fit for your research. If you are not sure, read the appropriate sections before making your selection.

☒ Life sciences ☐ Behavioural & social sciences ☐ Ecological, evolutionary & environmental sciences

For a reference copy of the document with all sections, see nature.com/documents/nr-reporting-summary-flat.pdf

Life sciences study design

All studies must disclose on these points even when the disclosure is negative.

Sample size	As given in the manuscript. Sample size estimates has been performed on previous experience to obtain statistical significance and reproducibility.
Data exclusions	As given in the manuscript. Only exclusions have been made in case of failed experimental procedures (based on pre-established criteria)
Replication	As given in the manuscript. All experiments underlying main conclusions of this study have been successfully replicated multiple times and corroborated by several models.
Randomization	As given in the manuscript. Samples and organisms were randomly allocated to experimental groups. No specific randomization protocol has been used. Mice were age- and sex matched.
Blinding	As given in the manuscript. No specific blinding was applied.

Reporting for specific materials, systems and methods

We require information from authors about some types of materials, experimental systems and methods used in many studies. Here, indicate whether each material, system or method listed is relevant to your study. If you are not sure if a list item applies to your research, read the appropriate section before selecting a response.

Materials & experimental systems

n/a	Involved in the study	
☐	☒	Antibodies
☐	☒	Eukaryotic cell lines
☒	☐	Palaeontology
☐	☒	Animals and other organisms
☒	☐	Human research participants
☒	☐	Clinical data

Methods

n/a	Involved in the study	
☒	☐	ChIP-seq
☐	☒	Flow cytometry
☒	☐	MRI-based neuroimaging

Antibodies

Antibodies used	As given in the manuscript
Validation	As given in the manuscript and using knockout or/and siRNA treated cell lines/samples.

Eukaryotic cell lines

Policy information about cell lines

Cell line source(s)	As given in the manuscript
Authentication	As given in the manuscript
Mycoplasma contamination	All used cell lines were tested negative for contamination
Commonly misidentified lines (See ICLAC register)	None of commonly misidentified cell lines has been used.

Animals and other organisms

Policy information about studies involving animals; ARRIVE guidelines recommended for reporting animal research

Laboratory animals	As given in the manuscript
Wild animals	n.a.
Field-collected samples	n.a.
Ethics oversight	As given in the manuscript: maintenance and animal experimentation were in accordance with the local ethical cimmittee in compliance with the European legislation on care and use of laboratory animals.

Note that full information on the approval of the study protocol must also be provided in the manuscript.

Flow Cytometry

Plots

Confirm that:

- ☐ The axis labels state the marker and fluorochrome used (e.g. CD4-FITC).
- ☐ The axis scales are clearly visible. Include numbers along axes only for bottom left plot of group (a 'group' is an analysis of identical markers).
- ☐ All plots are contour plots with outliers or pseudocolor plots.
- ☐ A numerical value for number of cells or percentage (with statistics) is provided.

Methodology

Sample preparation	Transiently transfected CHO cells were digested with accutase (ThermoFisher, A1110501) and washed twice with cold NaAc acidic buffer (0.3 M NaCH3COO pH 4.8, 150 mM NaCl, 1 mM MgCl2, 1 mM CaCl2). Then, cells were incubated with 20 µg /ml AF647-LDL in acidic buffer for 20 min on ice followed by 3 washes with NaAc acidic buffer and fixed with 2% paraformaldehyde in PBS.
Instrument	CytoFLEX flow cytometer (BECKMAN)
Software	CytoExpert 2.0
Cell population abundance	No cell sorting was performed
Gating strategy	No extensive gating strategy was taken.

☐ Tick this box to confirm that a figure exemplifying the gating strategy is provided in the Supplementary Information.
